# Changing concepts in anaesthesia for day care surgery

**DOI:** 10.4103/0019-5049.72635

**Published:** 2010

**Authors:** SS Harsoor

**Affiliations:** Editor, Indian Journal of Anaesthesia, No 21, 2nd Cross, Kirloskar Colony, Basaveshwar Nagar, II Stage, Bangalore - 560 079, India. E-mail: harsoorss@hotmail.comeditorija@yahoo.in

The global economic constraints and increasing financial awareness of 1970s led to the increase in the incidence of ambulatory surgery. Recent advances in medical technology, anaesthesia and pain management have allowed a huge expansion of this modality of care with a consequent reduction in the need for hospitalization. These facilities of ambulatory anaesthesia may be attached to main hospital itself, or office based or free standing. The convenience and low overhead costs continue to attract more surgeries to be conducted in an ambulatory setting. Several experts predict that in the years to come, nearly 80% of all surgeries performed in the United States will be on an ambulatory basis. But the standard of anaesthesia care is the same as that applicable to hospital-based surgeries, even for the most minor surgeries. A backup emergency care, either at same place or at a nearby hospital, must be available.

The ambulatory surgical practice offers several advantages to patients, doctors, and insurance companies, and the cost is expected to be 25–75% lesser than that of a similar inpatient procedure. Studies have reported that the total cost of anaesthesia and recovery using spinal anaesthesia is similar to that for general anaesthesia (GA).[1] But for the successful conduct of anaesthesia, careful patient selection, type of surgeries based on the facilities available, appropriate preparation of patient and planning are essential. Federated Ambulatory Surgery Association (FASA) has observed that there is no significant relationship between pre-existing diseases and incidence of postoperative complications in an ambulatory setting. Even the extremes of age are not deterrent for ambulatory practice, provided proper attention is paid to discharge planning. Children are excellent candidates for ambulatory surgery as it provides minimal separation from parents and minimal exposure to potentially contaminated hospital environment.

## PRE-ANAESTHETIC EVALUATION

In addition to reducing anxiety about the surgery and anaesthesia, pre-anaesthetic evaluation allows the anaesthesiologist to identify potential medical problems in advance, determine their aetiology, and if indicated, initiate appropriate corrective measures. The goals must be to resolve preoperative problems well in advance, thereby minimising the numbers of both cancellations and complications.

Presently, there are several commonly used approaches to screening patients for ambulatory surgery. These include facility visit or office visit prior to the day of surgery or preoperative screening with visit on the morning of surgery, among others. Each system has its own advantages and disadvantages. Ideally, the patient should visit Pre Anaesthetic Evaluation (PAE) clinic and have the assessment to avoid last minute cancellations. Certain disorders such as undiagnosed OSA may be relatively common in an ambulatory surgical population as these patients demand a vigilant perioperative care.[2] Basic minimum laboratory investigations can be conducted during the above period and appropriate counselling provided to the patient.

The patients are allowed clear liquids up to 2 hours before surgery, without increasing residual gastric volume. Administration of H_2_ blockers and metoclopramide can reduce both residual gastric volume and acidity. The intake of oral fluids may actually dilute gastric secretions and stimulate gastric emptying, resulting in lower residual gastric volumes.[3]

The patients should take all chronic oral medications up to 1 hour before the procedure. Special care must be exercised to continue beta-blockers and calcium channel blockers on the day of surgery. Continuing angiotensin converting enzyme (ACE) inhibitors and angiotensin receptor blockers may increase the likelihood of intraoperative hypotension but it will respond to simple treatments without any apparent adverse outcomes.[4] As most ambulatory surgical procedures present low bleeding risk, the current attitude in ambulatory setting is to maintain aspirin therapy and possible antiplatelet drug inhibitors throughout the perioperative period.[5] Further, it is suggested that in all surgical situations under ambulatory setting, antiplatelet therapy should be maintained, and if bleeding is likely to threaten either the patient’s life or the success of the surgical procedure, the discontinuation protocol must be established in conjunction with the cardiologist and the antiplatelet therapy resumed as soon as possible. Bridging with low–molecular-weight heparins is not recommended.[6]

Preoperative sedation, amnesics and anxiolytic drugs can be administered safely without any clinically significant delay in recovery times even after short ambulatory procedures.[7] Even drugs such as ketamine are used for premedication effectively in mentally disabled patients undergoing major dental surgery, without any increase in the incidence of side effects.[8]

## TECHNIQUES

In ambulatory practice, total intravenous anaesthesia (TIVA) provides advantages for all short surgical procedures and for ENT and ophthalmic surgeries, as even after prolonged infusion, children have a rapid recovery time, no agitation or other behavioural disorders.[9] Use of anaesthetic adjuvants like Dexmeditomidine are known to minimise sevoflurane-associated emergence agitation (EA) and postoperative pain in paediatric ambulatory surgery.[10] Newer technology is always of benefit to ambulatory anaesthesia practice. Ultrasound-guided interscalene and supraclavicular blocks are used effectively and safely for ambulatory shoulder arthroscopy compared to landmark based nerve blocks.[11] Newer spinal anaesthetic techniques for common ambulatory procedures highlight the success of combining subclinical doses of local anaesthetics and intrathecal opioid adjuncts.[12] The neuraxial block with shorter acting local anaesthetic agents, specific to the expected duration of surgery, may provide superior recovery profiles in the ambulatory setting.[13] Isobaric prilocaine has a longer duration of action than an equal dose of lignocaine and may be an alternative drug for spinal anaesthesia when intermediate or short duration of action is needed. Transient neurological symptoms (TNS) can occur after spinal anaesthesia with isobaric prilocaine also. Though ropivacaine has not shown benefits over spinal anaesthesia with bupivacaine, the “walk-in, walk-out” spinals with an extremely low dose of lignocaine and opioids for gynaecological laparoscopy have generated a concept of selective spinal anaesthesia.[14] Gynaecological laparoscopy done under spinal with lignocaine 10 mg + sufentanil 10 μg compared with GA with desflurane and N_2_O showed that with spinal anaesthesia, patients can walk from the operating room table to a stretcher on completion of surgery and recovery time was similar to that of the desflurane group.[15] Spinal anaesthesia in the outpatient is characterised by rapid onset and offset, easy administration, minimal expense, and minimal side effects or complications and offers advantages for outpatient lower extremity, perineal, and many abdominal and gynaecological procedures.[16]

The development of small-gauge, pencil-point needles are responsible for the success of outpatient spinal anaesthesia with acceptable rates (0–2%) of postdural puncture headache (PDPH), and compared with peripheral nerve blocks, spinal anaesthesia has a more predictable offset.

## CONCERNS FOLLOWING SPINAL ANAESTHESIA

### Postdural puncture headache

Use of small 25- or 27-gauze pencil point needle offers a very low incidence of PDPH. Contrary to perception, early ambulation does not appear to play a role in PDPH.

### Transient neurological symptoms

The aetiology of TNS remains obscure though lignocaine has been implicated often. Interestingly, ambulatory surgery has been identified as a contributory factor. The incidence is higher in patient on lithotomy or knee arthroscopy position. Apart from lignocaine, even procaine and mepivacaine also have higher incidence of TNS, and bupivacaine has the lowest incidence.

### Postoperative nausea and vomiting

The incidence of postoperative nausea and vomiting (PONV) following ambulatory anaesthesia varies between 2.2% and 4.6%, and there is a fivefold increase in PONV following GA compared to spinal anaesthesia. Since PONV is known to delay the patient discharge, a multimodal antiemetic treatment is more beneficial.

### Pain

Postoperative pain is the most significant complaint following ambulatory spinal anaesthesia. Factors associated with severe pain in the post anaesthesia care unit (PACU) include younger adults, ASA grade I patients, patients with a larger body mass index (BMI), prolonged duration of surgery and orthopaedic, urologic and plastic surgeries.[17] Here again, a concept of multimodal analgesic technique with combination local anaesthetics, non-steroidal anti-inflammatory drugs (NSAIDs), opioids has shown better results.

## CONTROVERSIAL ISSUES

Optimal evidence-based perioperative blood glucose control in patients undergoing ambulatory surgical procedures remains controversial. Therefore, the Society for Ambulatory Anesthesia (SAMBA) used the Grading of Recommendations, Assessment, Development, and Evaluation (GRADE) system for providing suggestions. In the absence of high-quality evidence, recommendations are made based on data from only inpatient surgical population, and management of diabetics.[18]

Whether to allow ambulatory patients to drink or withhold in the early postoperative period: resumption of oral intake and spontaneous voiding are no longer mandatory prerequisites for discharge after outpatient surgery. Mandatory drinking may in fact provoke nausea and vomiting.

Voiding prior to discharge: Blockade of sympathetic nerve supply to bladder and urethra may cause retention of urine. Micturation reflex returns on regression of subarachnoid block beyond S-3 level. Hence, in patients with low risk of retention, voiding before discharge appears unnecessary.[19]

## RECOVERY AND DISCHARGE PROCESS

The original Aldrete Score is useful in evaluating initial patient recovery after ambulatory anaesthesia, but the patients’ “home readiness” is better assessed with modified Post Anaesthesia Discharge Scoring system (PADSS). These scores are useful in allowing documentation of objective measurements of clinical recovery. However, the following simple recovery criteria are beneficial in routine clinical practice:

- simple psychomotor tests like memory and sensory motor coordination;
- recovery of motor and sensory functions: With spinal anaesthesia, it is generally accepted that motor and sensory functions return before recovery of sympathetic nerve system;
- two successive orthostatic MAP decrease of 10% or less;
- prior to ambulation, patients should have normal perianal sensation (S4–5), ability to plantarflex the foot, and proprioception of the big toe.

Following ambulatory anaesthesia, patients should be discharged home with an adult escort, who ideally will continue overnight supervision of patients’ recovery and patients should also have ready access to healthcare providers.

Hence, it is essential to define safe practice standards based on regional needs and economic considerations. The complications of anaesthesia outside the operating room still persist even in ASA status I patients and in accredited facilities. But adhering to practices such as documentation, guidelines preparation, equipment, standard monitoring, and collaboration with other institutional facilities, backup for the personnel in case of emergencies will enhance the safety, efficiency and reliability of office-based anaesthesia inside and outside the hospital.

Because outpatient anaesthesia is a breakaway from our traditional training, we are constantly being confronted with the need for change in our clinical practice patterns. It is obvious that there is much to learn about anaesthesia for ambulatory surgery.

## References

[1] Chilvers CR, Goodwin A, Vaghadia H, Mitchell GW (2001). Selective spinal anesthesia for outpatient laparoscopy: Pharmacoeconomic comparison vs general anesthesia. Can J Anaesth.

[2] Stierer TL, Wright C, George A, Thompson RE, Wu CL, Collop N (2010). Risk assessment of obstructive sleep apnea in a population of patients undergoing ambulatory surgery. J Clin Sleep Med.

[3] Hutchison A, Malltby JR, Reid CR (1998). Gastric fluid volumeand pH in elective patients. Part 1: Coffee or orange juice versus overnight fast. Can J Anaesth.

[4] Smith I, Jackson I (2010). Beta-blockers, calcium channel blockers, angiotensin converting enzyme inhibitors and angiotensin receptor blockers: Should they be stopped or not before ambulatory anaesthesia?. Curr Opin Anaesthesiol.

[5] Servin F (2007). Low-dose aspirin and clopidogrel: How to act in patients scheduled for day surgery. Curr Opin Anaesthesiol.

[6] Servin FS (2010). Is it time to re-evaluate the routines about stopping/keeping platelet inhibitors in conjunction to ambulatory surgery?. Curr Opin Anaesthesiol.

[7] Van Vlymen JM, Sa Rego MM, White PF (1999). Benzodiazepine premedication: Can it improve outcome in patients undergoing breast biopsy procedures?. Anesthesiology.

[8] Cortiñas M, Oya B, Caparros P, Cano G, Ibarra M, Martínez L (2010). Oral ketamine-midazolam premedication of uncooperative patients in major outpatient surgery. Rev Esp Anestesiol Reanim.

[9] Strauss JM, Giest J (2003). Total intravenous anesthesia. On the way to standard practice in pediatrics. Anaesthesist.

[10] Sato M, Shirakami G, Tazuke-Nishimura M, Matsuura S, Tanimoto K, Fukuda K (2010). Effect of single-dose dexmedetomidine on emergence agitation and recovery profiles after sevoflurane anesthesia in pediatric ambulatory surgery. J Anesth.

[11] Liu SS, Gordon MA, Shaw PM, Wilfred S, Shetty T, Yadeau JT (2010). A prospective clinical registry of ultrasound-guided regional anesthesia for ambulatory shoulder surgery. Anesth Analg.

[12] Nair GS, Abrishami A, Lermitte J, Chung F (2009). Systematic review of spinal anaesthesia using bupivacaine for ambulatory knee arthroscopy. Br J Anaesth.

[13] O’Donnell BD, Iohom G (2008). Regional anesthesia techniques for ambulatory orthopedic surgery. Curr Opin Anaesthesiol.

[14] Korhonen AM (2006). Use of spinal anaesthesia in day surgery. Curr Opin Anaesthesiol.

[15] Lennox PH, Vaghadia H, Henderson C, Martin L, Mitchell GW (2002). Small-dose selective spinal anesthesia for short-duration outpatient laparoscopy: Recovery characteristics compared with desflurane anesthesia. Anesth Analg.

[16] Urmey WF (2003). Spinal anaesthesia for outpatient surgery. Best Pract Res Clin Anaesthesiol.

[17] Jin FL, Chung F (1998). Postoperative pain- a challenge for anesthesiologist in ambulatory surgery. Can J Anaesth.

[18] Joshi GP, Chung F, Vann MA, Ahmad S, Gan TJ, Goulson DT (2010). Society for Ambulatory Anesthesia Consensus Statement on Perioperative Blood Glucose Management in Diabetic Patients Undergoing Ambulatory Surgery. Anesth Analg.

[19] Pavlin DJ, Pavlin EG, Fitzgibbon DR, Koerschgen ME, Plitt TM (1999). Management of bladder function after outpatient surgery. Anesthesiology.

